# The role of item size on choosing contrasted food quantities in angelfish (*Pterophyllum scalare*)

**DOI:** 10.1038/s41598-019-51753-1

**Published:** 2019-10-25

**Authors:** Luis M. Gómez-Laplaza, Laura Romero, Robert Gerlai

**Affiliations:** 1 Department of Psychology, University of Oviedo, Oviedo, Spain; 2 Department of Psychology, University of Toronto Mississauga, Mississauga, Canada

**Keywords:** Psychology, Animal behaviour

## Abstract

Comparative studies on quantity discrimination in animals are important for understanding potential evolutionary roots of numerical competence. A previous study with angelfish has shown that they discriminate numerically different sets of same-sized food items and prefer the larger set. However, variables that covary with number were not controlled and choice could have been influenced by variables such as size or density of the food items rather than numerical attributes. Here using a recently developed approach, we examined whether contour length of the food items affects choice in a spontaneous binary choice task. In Experiment 1, a contrast of 1 vs. 1 food item was presented, but the ratio between the size (diameter) of the food items was varied. In Experiment 2, numerically different food sets were equated in overall size by increasing the size (diameter) of the items in the numerically small sets. In both Experiments, subjects showed a preference for the larger sized food items with a discrimination limit. These results show that item size plays a prominent role in foraging decisions in angelfish. Experiment 3 placed numerical and size attributes of the sets in conflict by presenting one larger-sized food item in the numerically smaller set that also had smaller overall size (diameter) of food items. Angelfish showed no preference in any of the contrasts, suggesting that they could not make optimal foraging decisions when these attributes were in conflict. Maximization of energy return is central to optimal foraging. Accordingly, here item size was also found to be a key feature of the sets, although the numerical attributes of the sets also influenced the choice.

## Introduction

Numerical cognition, i.e., the capacity to perceive and discriminate differences in number, has been shown to be important for survival across species. In humans, some evidence indicates a connection between this ability in infants and later understanding of complex mathematical principles^1^. Thus, one of the key questions in animal cognition concerns the origins of numerical abilities, or the source of mathematical abilities, and how this emerged through evolution. In fact, the ability to discriminate relative differences in quantity is a trait that has been shown in a wide range of animal species^2^. One of the most commonly used tasks has been discrimination of food quantities, because identification of the larger quantity maximizes food intake and promotes evolutionary fitness^3^. However, numerical competence, i.e., the ability to utilize number/quantity-related cues when comparing larger vs smaller quantities, and the utilization of numerical information and non-linguistic quantification abilities have also been studied in other contexts including predator avoidance, shoaling and reproduction^2^. In this study we consider numerical competence in the foraging context. The optimal foraging theory (OFT) predicts that individuals should prefer the more profitable food item/set that maximizes energy gained (energy gain per unit handling time)^3^. A forager may be selected for its ability to assess the value of a set and thus choose optimally. Thus, when an individual encounters two food items/sets of the same type of food and at the same distance from the forager (i.e., the time required to reach one or the other is similar), in a relatively safe location (i.e., with no predators present), the theory predicts that individuals should choose the larger food item/set, at least initially, over the smaller to maximize food intake. However, with a few exceptions^4–6^, numerical capabilities of fish species, and the perceptual cues used to discriminate between sets, in this ecological context are practically unknown. Most studies examining food number/quantity discrimination has been performed with mammals^7–11^ and birds^12–15^, and very few also with reptiles^16,17^, amphibians^18,19^ and invertebrates^20^. In general, all these studies report preference for the larger food set. Very few of these laboratory studies attempted to address predictions of the optimal foraging theory (which needs to consider the costs of item capture and ingest and some environmental and morphological constraints), but were aimed to investigate diverse aspects related to numerical competence in animals and the factors that may affect it. These studies investigated the ability to discriminate sets with different numbers/quantities of items. They also investigated key feature(s) of the stimuli on which individuals rely for discrimination (e.g., whether on number per se or on other attributes of the stimuli that generally co-vary with number). A variety of species have been shown to respond consistently with Weber’s law^21^, in the sense that the discrimination between two quantities was found to be a function of the ratio between the number of items in the contrasted sets^22^. The larger the ratio of the larger to the smaller set, the better discrimination ability was found. The results also suggested the existence of two representation systems to account for quantity discrimination^22^: one of the systems proposed is independent of absolute size of the sets/items, but is based upon ratio, and hence is subject to Weber’s law. It is called the Approximate Number System (ANS), and it is thought to operate for discriminating sets in the large number (>4 elements) range, although can also represent the entire range of numbers^23^. The other system, proposed specifically for the representation of the small number range (sets composed of ≤4 elements), the Object File System (OFS), is believed to allow animals to distinguish the contrasted sets based upon absolute numerical difference (rather than ratio). The OFS, however, rather than being a proper quantity representation system, is conceived as a mechanism of visual attention, therefore having a limited capacity^24^. These underlying mechanisms for discrimination seem to be of adaptive value and are apparently shared across vertebrates, which suggest an evolutionary ancient origin.

Although some findings suggest that different species are able to perceive numerical size differences between sets^25–30^, number usually co-varies with other features of the stimuli (often called non-numerical continuous variables), which could also constitute the basis for the discrimination^31^. What cues animal species would prefer to use when choosing between contrasted item sets and whether these cues are numerical or non-numerical have been frequently debated in the literature^32,33^. Recently, we have shown that angelfish are able to spontaneously (without specific training) select the numerically larger food set in a dichotomous choice task^6^. However, as in other studies of similar nature, the number of discrete food pieces of equal physical size in a set covaried with the quantity of food. Thus, we could not tell whether the choice made by our experimental angelfish was based upon numerical or non-numerical attributes of the presented food item sets (e.g., contour length of the items, cumulative surface area, density), or perhaps both. Consequently, more information is needed regarding the extent to which non-numerical variables such as the contour length (related to size) of the items in the sets influenced discrimination. In a social context, where angelfish had to discriminate between numerically different shoals (groups) of conspecifics, we have already obtained evidence demonstrating the importance of continuous variables, a finding also obtained with other fish species^34–36^. The variables we found to play roles were the level of activity, size, and the density of the presented stimulus fish^37–39^.

**Figure 1:**
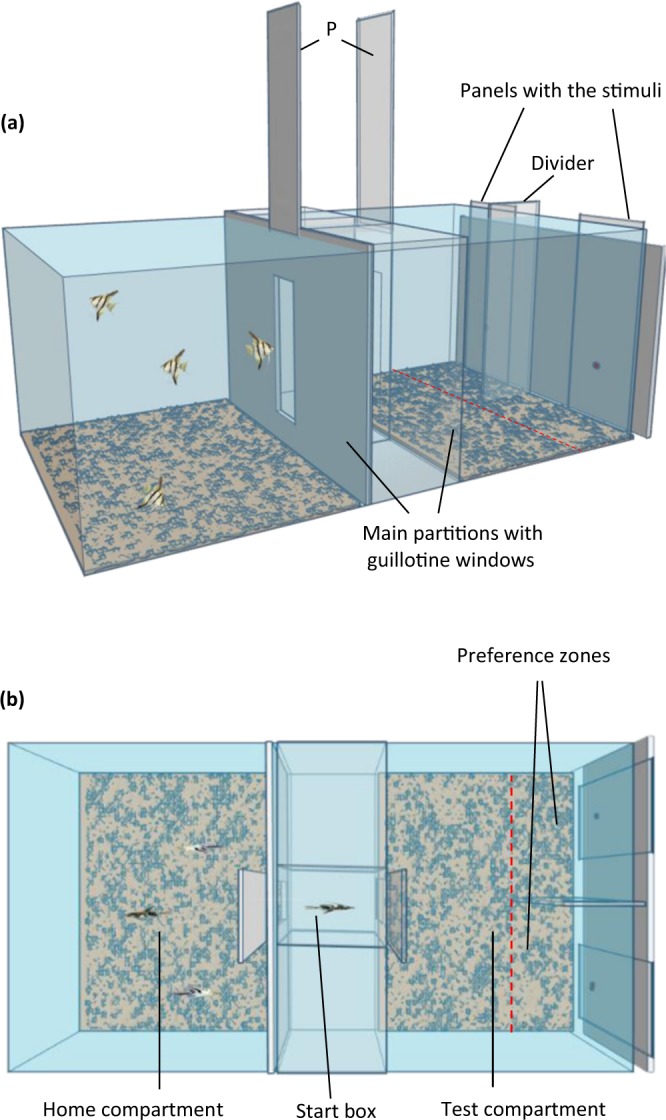
Schematic representation of the experimental aquarium including partitions and panels. **(a)** Side view showing the main partitions (transparent and white opaque), with the guillotine windows, that delimited the compartments. The transparent plastic divider that delimited the preference zones and the panels where the stimuli were presented are also indicated. P: refers to the panels that the experimenter could raise or lower to open or close the guillotine windows. **(b)** Top view showing the home and test compartments separated by a smaller middle compartment whose central part constituted the start box. The middle compartment is represented devoid of sand (although it had it) for greater clarity. The preference zones are also indicated (dashed line).

The current research represents one of the few attempts whose goal is to examine food quantity discrimination in fish, with a specific aim to provide a systematic investigation of the influence of the size of the food items in the discrimination of food sets. For comparative purposes, the study also aims to acquire information about the potential underlying mechanisms used by angelfish in solving the task, a topic of growing interest among ethologists and comparative psychologists. In several species^16,17,40–44^, including the only systematic study of this kind with fish^4^, difference in the size of the food items has been reported as one of the most prominent variables to be used to discriminate between food sets. Here, we manipulated the size (contour length) of the individual food items to explore whether this feature of the stimuli could influence the incentive value of, and thus the preference for, sets containing larger-sized items. To achieve our objectives, we employ the freshwater angelfish, *Pterophyllum scalare*, as our model organism. The angelfish is a social cichlid fish species that exhibit characteristic group feeding habits. Native of the Amazon basin, it is widely distributed over a vast area, living in habitats that are apparently dynamic in terms of structure and food type and availability^45^. Briefly, the ecology of the angelfish predicts a generalized, rather than specialized, foraging strategy in this species. Furthermore, laboratory studies have found juvenile angelfish to possess complex learning abilities^46–48^. Angelfish, as a highly social species, has also been shown to be able to discriminate shoals of conspecifics differing in number^49–52^, and also to remember the location of the larger vs the smaller shoals^53–56^. All these behavioural and cognitive traits make the angelfish an ideal species for studying food quantity discrimination. We also emphasize that in our current study we utilize a recently developed new approach^6^ that minimizes human handling and isolation induced stress (Fig. 1). The methodology as well as the conceptual aspects of this approach, we argue, will be useful for comparing numerical abilities among multiple species.

**Figure 2:**
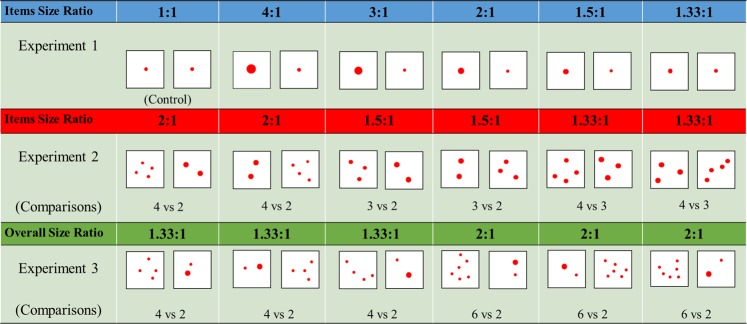
Examples of the ratios tested in each of the experiments and the corresponding numerical comparisons. Food items are not to scale.

The main hypothesis of our study is that if angelfish are able to perceive size differences between food items, in accordance with the optimal foraging theory, they should prefer the larger food item to maximize energy intake and minimize cost of searching and hunting for food. In addition to testing this hypothesis, importantly, we also aim to determine whether performance of experimental angelfish depend upon the ratio between the size of the items and what the ratio limit of discrimination is. Furthermore, we also investigate how angelfish respond when the food item size and number are conflicted to explore the relative importance of these factors and whether angelfish respond optimally according to total food intake (see Fig. 2).

## Results

**Figure 3:**
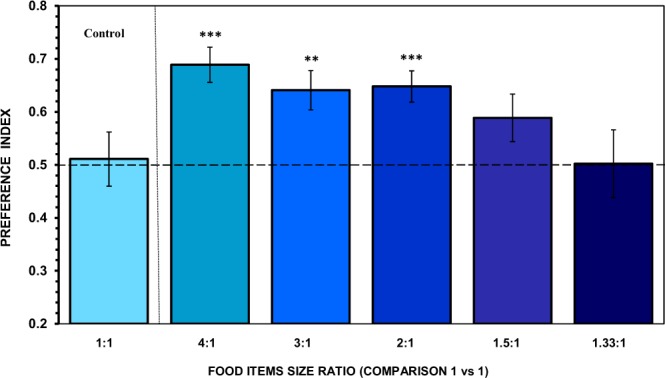
Choice of angelfish as a function of the ratio of the larger item contour length to the smaller item contour length. Mean ± s.e.m. proportion of time (preference index) spent by test fish in the preference zone close to the larger-sized food item (contour length). The contrast was 1 vs 1 food item. The control treatment (equal food item sizes) is also included. Values above 0.5 indicate a preference for the larger item size. Significant departure from the null hypothesis of no preference is indicated by asterisks: ***P < 0.005, **P < 0.01.

In the control treatment, no significant difference relative to a random choice was found. The fish did not exhibit significant bias for either side of the aquarium where the stimuli were presented, and did not spend more time in one or the other preference zones (t(11) = 0.212, p = 0.836; Fig. 3 control bar). All other behavioural parameters measured (first choice, frequency of entries and latency to enter the preference zones) also confirmed the absence of preference for one versus the other of the same-size food items (Table 1). These results indicate that the apparatus and procedure is devoid of unknown confounds leading to side bias.

**Table 1:** Performance of angelfish (N = 12 in each comparison) when faced with the different ratios (Experiment 1, 2, and 3) and numerical contrasts (Experiment 2 and 3).

Items size ratios	First choice (out of 12 fish)^a^			Frecuency of entries^b^				Latency^c^			
	Larger food item	Smaller food item	Binomial test	Larger food item	Smaller food item	t test		Larger food item	Smaller food item	t test	
						t_11_ value	Probability			t_11_ value	Probability
**Experiment 1**											
1: 1	5	7	*P* = 0.774	3.92 ± 0.62	3.67 ± 0.38	0.390	*P* = 0.704	44.17 ± 11.94	26.83 ± 10.87	0.878	*P* = 0.399
4: 1	11	1	*P* = 0.006	6.08 ± 0.51	4.75 ± 0.87	2.969	*P* = 0.018	5.08 ± 2.94	69.17 ± 12.39	5.413	*P* < 0.001
3: 1	10	2	*P* = 0.039	4.00 ± 0.43	2.83 ± 0.32	3.189	*P* = 0.009	11.33 ± 6.36	48.75 ± 9.51	2.584	*P* = 0.025
2: 1	11	1	*P* = 0.006	5.50 ± 0.56	3.50 ± 0.40	4.195	*P* = 0.001	6.67 ± 5.22	48.42 ± 15.65	4.198	*P* = 0.001
1.5: 1	11	1	*P* = 0.006	7.50 ± 1.22	4.92 ± 0.96	2.466	*P* = 0.031	6.08 ± 2.25	67.08 ± 15.37	4.353	*P* = 0.001
1.33: 1	9	3	*P* = 0.146	4.50 ± 1.18	4.42 ± 0.83	0.076	*P* = 0.940	21.83 ± 10.75	51.58 ± 22.37	1.310	*P* = 0.217
**Experiment 2 (Contrasts)**											
2: 1 (4 vs. 2)	11	1	*P* = 0.006	4.83 ± 0.67	3.67 ± 0.57	1.629	*P* = 0.131	7.33 ± 1.80	74.50 ± 12.90	4.369	*P* = 0.001
1.5:1 (3 vs. 2)	10	2	*P* = 0.039	4.83 ± 0.53	4.67 ± 0.86	0.198	*P* = 0.847	10.83 ± 4.37	47.17 ± 9.57	2.758	*P* = 0.019
1.33: 1 (4 vs. 3)	8	4	*P* = 0.388	4.58 ± 0.42	6.00 ± 0.64	1.880	*P* = 0.087	13.00 ± 4.82	28.83 ± 9.47	1.233	*P* = 0.243
**Experiment 3 (Contrasts)**											
1.33: 1 (4 vs 2)	9	3	*P* = 0.146	5.75 ± 0.81	3.67 ± 0.43	2.046	*P* = 0.065	19.33 ± 9.98	59.83 ± 17.89	1.846	*P* = 0.092
2: 1 (6 vs 2)	8	4	*P* = 0.388	6.83 ± 0.77	5.67 ± 0.92	1.196	*P* = 0.257	11.58 ± 4.12	31.08 ± 12.79	1.037	*P* = 0.322

*Note*. Subjects were tested individually. Descriptive statistics includes means ± s.e.m. The tests used to compare the scores are also included. ^a^Number of fish whose first choice was one or the other stimulus set. ^b^Frecuency, number of times that subjects entered to the preference zones. ^c^Latency to enter the preference zone near one or the other stimulus set.

When subjects were confronted with a choice between two food items of different size (diameter), the response of angelfish varied with the ratio between the contour length (size) of the items (Fig. 3). They exhibited a preference for the larger item, and spent significantly more time than expected by chance in the preference zone close to it when the ratio was 2:1 or greater (one-sample t test, with Holm-Bonferroni correction: 4:1, t(11) = 5.667, p < 0.001, *d* = 1.63; 3:1, t(11) = 3.804, p = 0.009, *d* = 1.10; 2:1, t(11) = 5.035, p = 0.004, *d* = 1.45), but when the ratio was 1.5:1 or lower, no significant preference was found (1.5:1, t(11) = 1.961, p = 0.076; 1.33:1, t(11) = 0.029, p = 0.977). One-way ANOVA revealed a significant difference in the magnitude of the preference between the five contrasts (F(4,55) = 2.702, p = 0.040, ${\eta }_{p}^{2}$ = 0.16), which was mainly due to the greater response when the ratio was 4:1 compared to performance when it was 1.33:1 (p = 0.030; Tukey HSD tests). Linear regression analysis indicated that the magnitude of the preference increased with increasing ratio. Although the increase did not reach the level of significance (F(1,3) = 6.231, R^2^ = 0.567, p = 0.088), we observed that as the contour length ratio was decreasing, discrimination (i.e., preference) became increasingly difficult. This preferential response for the larger food item was also shown in the other parameters measured (Table 1). We emphasize that the analysis of these other parameters revealed a significant discrimination of food items also differing with a 1.5:1 contour length ratio. Thus, the results indicate that angelfish can see which of the contrasted food items is larger, will swim towards it, and prefer staying close to it as long as the contour length ratio reaches or exceed 1.5:1.

**Figure 4:**
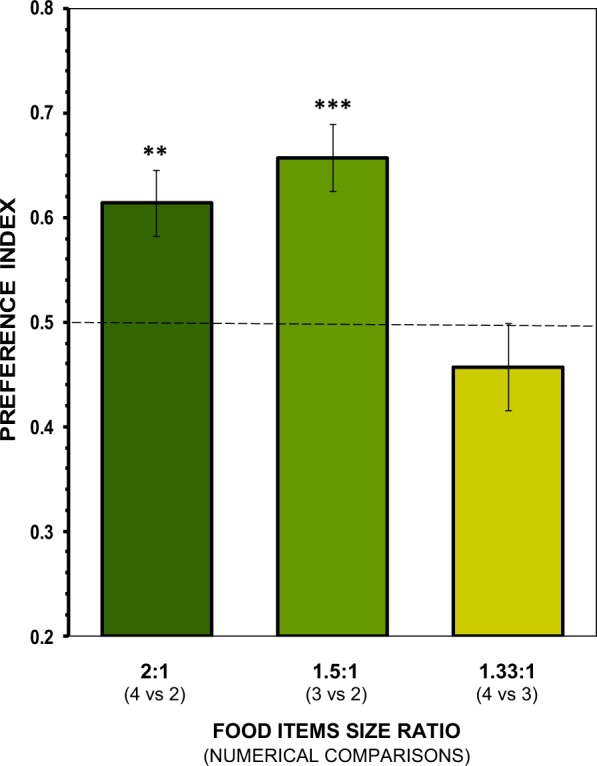
Choice of angelfish as a function of the ratio between the food item-size in the numerically larger set to the smaller set, when the overall contour length of food in the sets was equalized. Mean ± s.e.m. proportion of time (preference index) spent by test fish in the preference zone close to the numerically smaller sets with the larger-sized food items (contour lengths). Values above 0.5 indicate a preference for the larger item sizes. Significant departure from the null hypothesis of no preference is indicated by asterisks: ***P < 0.005, **P < 0.01.

In Experiment 2, when contrasting numerically and item-size-wise different food item sets while keeping the total contour length of the contrasted item sets constant results showed that angelfish preferred the sets with the larger-sized food items and not the numerically larger sets, when the food item contour length ratio was of 1.5:1 or greater (Fig. 4). The preference was significantly above chance for the 2:1 item contour length ratio (t test with Holm-Bonferroni correction, t(11) = 3.559, p = 0.008, *d* = 1.03) and for the 1.5:1 item contour length ratio (t(11) = 4.868, p = 0.0024, *d* = 1.41), but not for the 1.33:1 contour length ratio (t(11) = 1.012, p = 0.333). One-way ANOVA revealed a significant difference among the contrasts in the magnitude of the preference for the sets with the larger food items (F(2,33) = 8.609, p = 0.001, ${\eta }_{p}^{2}$ = 0.34), and Tukey HSD test indicated that this was due to the significantly greater preference for the sets with the larger food items in the 2:1 and 1.5:1 contour length ratios as compared to 1.33:1 size ratio (all p ≤ 0.011). Therefore, we conclude that when the two attributes (food item size versus item number) are conflicted, angelfish decide based upon food item size. The analysis of other behavioural parameters (Table 1), also indicated that generally item size took priority over number of items when item contour length ratio was of 1.5:1 and greater, a limit that is coincident with that found in Experiment 1.

**Figure 5:**
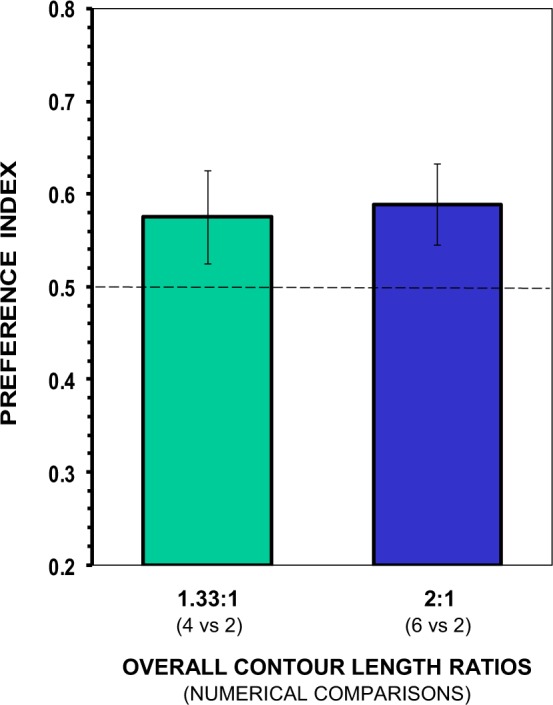
Choice of angelfish as a function of the overall ratio of contour length of food items of the sets, when the numerically smaller set had a larger-sized food item and less overall contour length of the items than the larger set. Mean ± s.e.m. proportion of time (preference index) spent by test fish in the preference zone close to the smaller set containing a food item that doubled the size of the others. Values above 0.5 indicate a preference for the set with the larger-sized food item. No significant departure from the null hypothesis of no preference was found.

In Experiment 3, the test fish chose in a manner that was statistically indistinguishable from chance (Fig. 5). They showed no significant preference for the location of the set with a larger food item when the overall ratio of item sizes (contour length of the items) between sets was 1.33:1 (t(11) = 1.492, p = 0.164), or when that overall ratio was 2:1 (t(11) = 1.998, p = 0.071). Interestingly, in both contrasts angelfish showed an apparent preference for the set with smaller total contour length but having one large-sized item, but this preference did not reach statistical significance. Analysis of the other behavioural parameters, including first food set selected, latency to enter the preference zones and frequency of entries confirmed this observation and showed no significant departure from chance (all p > 0.05; Table 1).

## Discussion

In this study, we examined the relative role that item contour length plays in food quantity discrimination. This is an important question given the existing and continued debate between those that claim that discrimination between sets differing in number of items can be strictly based on number (i.e., discrete numerosities), and those that claim that number cannot be isolated from the concomitant continuous variables of the stimuli and, therefore, quantity discrimination may depend upon non-numerical, continuous attributes of the contrasted item sets^33^. The current experiments now provide evidence for both: we demonstrate that angelfish relies on differences in the sizes of the food items, as well as on numerical information when choosing between sets of food items.

A previous study demonstrated that angelfish presented with same-sized food items could discriminate between food sets differing in quantity (i.e., number of items) as much in the large (>4 food items) as in the small (≤4 food items) number range^6^. In that study, continuous variables were not controlled, and, instead of number, experimental angelfish could rely on such variables^6^. For example, more food items also were denser, had greater overall contour length, and occupied more cumulative surface area in the presentation panel than numerically fewer food items did (i.e., we had a congruent condition between number and continuous variables).

Here, also with stimuli constantly in view, angelfish could compare the sets directly on the basis of perceptual features. According to OFT^3^, to maximize its feeding efficiency, a forager should select the set with the highest net rate of return. In agreement with this prediction, we found angelfish to prefer, the larger contour length food item (the richer set) to the smaller sized one (the poorer one) in the absence of predation risk. In contexts other than foraging, quantity discrimination studies have also demonstrated that the size of the items, including contour length^57^, is an important variable affecting discrimination in diverse species^30,58^. Similar results have also been found in one fish species, the guppy (*Poecilia reticulata*)^4,5^ in a foraging context. Size-dependent prey selection has also been shown to be an important factor affecting foraging^59,60^, although in these studies foragers do not always select the larger prey. Calculation of energy benefits and costs (e.g., prey capture) of different actions as well as diverse constraints on the forager and environment, may result in smaller preys providing the greatest energy return. Our study, however, uncomplicated by the above factors, because prey handling time, time pursuing prey, encounter rate, capture success, etc., are not expected to influence choice. As a result, we expect preference for the stationary and “nibbleable” food items to be shifted towards the larger-sized food items.

A salient influence of the larger food item size in the decision-making process has been reported in chimpanzees^40,43^ and cotton-top tamarins^42^, and an effect of the cumulative surface area was found in monkeys^31^. Likewise, a preference for the larger food prey have been shown in lizards^16^, tortoises^17^ and also in cuttlefish^20^. In the studies with reptiles^16,17^, the operation of the Approximate Number System (ANS) was proposed. In these studies, subjects had to select between two food items of different size, and just like in the current study, the response was found to be size ratio-dependent (i.e., discrimination became more difficult as the ratio of the larger to the smaller size item decreased). In most of the above studies, size was measured as surface area, total surface area, or volume occupied by the items, and the differences between the size of the items, so measured, were generally smaller than those employed in the current study. In a study with human infants, discrimination of contrasted sets was found to be dependent upon both contour length^57^ and also surface area^61^ (not surprising given that contour length and surface area are related to each other). Importantly, however, unlike in our current study, these authors found that infants did not rely on numerical information^57^.

In our current study, we demonstrate that the strength of preference exhibited by angelfish towards the preferred (larger) food item set is dependent upon the ratio between the contour lengths of the contrasted food item sets. That is, we found the preference exhibited by angelfish to conform to Weber’s law, a typical signature of the ANS. We found the limit of size discrimination ratio to be 1.5:1. Interestingly a similar ratio limit has been previously found in angelfish in a foraging context^6^, as well as in a social context^50^. A food-size discrimination ratio was also demonstrated in guppies with a performance following Weber’s law^4,5^. However, in these latter studies, size of the items was calculated on the basis of their surface area, and guppies proved able to discriminate much smaller size differences than our angelfish. The limit we found in angelfish could be the result of motivational processes. i.e., below a certain level of difference, it does not matter which food item the animal chooses, as both offer similar fitness advantages. It may also be due to inability to perceive different sizes apart below a certain level of difference. The ratio dependency of performance found across a variety of vertebrates from fish of the above studies to reptiles^16,17^ and chimpanzees^43^ indicate that such visual cue as the size of the items may play a fundamental role in the discrimination of food quantities. This cue may indicate amount of food available to capture or consume, and may allow the animal to maximize foraging success.

The results of Experiment 2 support the later hypothesis, and demonstrated that the size of food items as opposed to their number is a feature of the stimuli upon which angelfish based their choice. Angelfish preferred the sets with the larger-sized food items, and again, as in Experiment 1, the limit of contour length ratio that angelfish could discriminate was found to be 1.5:1. Thus, with ratios 1.5:1 and greater the significantly shorter latencies and number of fish choosing first the set with the larger items indicate that differences in size positively influenced stimulus discrimination. With ratios below 1.5:1 the two sets of stimuli were non-distinguishable for angelfish or had equal relevance as suggested by Experiment 1. When difference between sets are not readily apparent, OFT predicts that the forager should sample the different sets to gain better information about the sets^3^. We found angelfish to visit (sample) both food sets with equivalent frequency, suggesting that these experimental fish were attempting to evaluate the sets and their potential value.

It is also important to consider the motivational state of experimental fish making the choice. For example, Yang and Chiao^20^ found that cuttlefish preferred one large live prey over two small live preys when the cuttlefish were hungry, but chose the two smaller preys when they were satiated. The results indicated that motivational factors (satiation levels) clearly affected the choice. In the context of OFT the relationship between motivational constraints and selection of prey size has been well documented in fishes^62,63^. Findings generally indicate that the state of satiation affects prey selection, as larger prey represents a greater energy gain. In our study, although differences in motivational state related to hunger could have existed, this may be an unlikely factor since hunger levels were standardized, food was not scarce, and fish were well fed during the habituation phase. Thus, the failure in the discrimination below 1.5:1 size ratio was likely due to perceptual constraints.

The preference for larger contour length food items as opposed to more numerous food items we found here with angelfish is similar to findings obtained with guppies^4^, chimpanzees^40,43^ and cotton-top tamarins^42^ in quantity discrimination studies. There may be several reasons for this choice common to a variety of species. Large-sized food items are easier to monopolize than more numerous small items, which may be a deciding factor in species that are social, hunt or gather food in groups. Angelfish is a shoaling species that explores environments searching for food, and presumably compete for resources while attempting to avoid abundant predators^64^. A cognitive mechanism that allows discrimination of the larger-sized food items in a patch should provide advantage in the context of foraging efficiency. Preference for larger-sized food items may also be advantageous from the perspective of predation risk, as the larger sized food item may be obtained faster than numerous smaller food items leading to reduced level of exploratory activity and reduced area of exploration thus minimizing the chance of encountering a dangerous predator. Although not all species of animals must follow this pattern, selection for larger-sized items in a competitive situation may be advantageous in a variety of species. However, as discussed above, the nature of the food item, i.e., whether it is a mobile prey, or a stationary food item, may also make a difference. For example, the body size of the predator relative to that of the prey (e.g., the ratio between the width of a prey and the diameter of the mouth of the predator), determines choice of prey eaten^62^.

The finding that number of food items did not play a primary role in food quantity discrimination, agrees with findings in some other studies^15,65,66^, but it does not imply that number is not a salient dimension that can control discrimination under similar conditions. For example, a study with treefrogs^30^ in which the experimental frogs had to choose between 4 vs 2 vertical bars (simulating vegetation) having the same overall surface area or convex hull, the frogs based their choice on the number of bars demonstrating that numerical cues formed the basis of the discrimination. Likewise, using edible stimuli, rhesus monkeys^67^ and domestic horses^68^ when having to choose between two opaque containers, baited sequentially with a different number of apple slices or artificial apples, but containing the same total volume, chose the container with the greater number of elements, indicating that they used number as the dominant cue. Other studies using different tasks and stimuli, have also demonstrated that number can be a salient dimension of a set of elements on which individuals base their discrimination decisions^31,44,69^. In addition to variations in stimuli presentation and contrasts tested in the different studies, it appears that when food items are visible, individuals try to maximize the relative size of the individual items rather than the number of items.

If angelfish focusses on item contour length alone, the subjective reward value of the larger items may affect its choice in a way that is suboptimal from the perspective of the total amount of food attained. In experiment 3, we contrasted sets of items that differed in visible contour length and found that angelfish did not show a statistically appreciable preference for either of the two contrasted sets. This, at first sight, appears to be in contradiction to the common assumption that animals should prefer the larger food item (related to food amount), but here the set with the fewer number of items contained one item that was physically larger. As previously we found angelfish to be able to distinguish numerically different food item sets^6^, and also found that food item size, rather than food item number, dominates the decision-making process of angelfish (Experiment 2), we conclude that in Experiment 3, the presence of a single large-sized food item abolished the preference for the numerically larger other food item sets, and surprisingly, also abolished the expected preference for the sets with the larger overall contour length, a conclusion that is also in accordance with findings obtained with dogs using a similar experimental approach^66^. Our reasoning is also supported by findings obtained with another fish species, guppies^4^, although guppies were presented with several trials for each contrast tested. Under such conditions, guppies chose the set with the larger-sized food item showing that they chose even more suboptimally than angelfish did (i.e., relied more on food item size, measured as the surface area) as far as maximizing obtainable food amount is concerned. Similar preference for smaller sets containing the larger-sized food item has also been found in nonhuman primates^40,42,43,70^. The results of Experiments 1 and 2 are compatible with the notion that angelfish use item contour length as a key factor driving their choice (“go for larger-sized food item”). However, the results of Experiment 3 contradict this hypothesis. Experiment 3 demonstrated that experimental angelfish, in addition to food item contour length, also took into account the number of items presented in the sets. Thus, it appears that, both food item contour length and the number of food items are important features experimental angelfish pay attention to. We hypothesize that this may be because angelfish, being a social forager, must compromise between maximizing food intake and minimizing competition for food. Since in nature size or number may not be universal indications of profitability, and the environmental conditions can be changing, evaluating, or integrating, different variables can be an effective means of decision making for angelfish and other species. Conditions in the field are very different from the laboratory environment with predation risk constraints, exploitation rates in the patches, live stimuli (that allow only certain manipulation), quality of the food, etc. Therefore, full information of the patches is complicated and animals have to combine various factors to optimize decisions to ensure a net gain. The present study provides evidence for sensitivity of angelfish to differences in the size of food items, with numerical information playing also a role. These factors, i.e., prey size and abundance have also been shown to affect selection of the most profitable prey/patch under more natural conditions in other fish species^71^. Since contour length is correlated with area, brightness and some other variables, it could be that the discrimination had been affected by any or all of these other variables. Therefore, another task for future studies is to investigate what other features, including density of the items, convex hull, surface area, colour, or shape that may co-vary with the presented food item number can influence the optimization of angelfish food choice. The similarities of the quantitative abilities of angelfish with those of other species studied that we found here, suggest that the underlying numerical systems may be evolutionarily conserved, although the possibility of parallel evolution, i.e., evolutionary analogies, in this cognitive trait cannot be disregarded.

## Methods

### Subjects and housing conditions

As in previous studies^6,49,53,54^, juvenile angelfish of 3.0–3.3 cm standard length were obtained from a local commercial supplier and were acclimatized for a minimum of two weeks before experimental procedures began. The fish were kept in two laboratory glass aquaria (60 × 30 × 40 cm, length × width × depth) in groups of 18–20 individuals per aquarium. The rationale for using juveniles is explained in detail elsewhere^6,49^. Briefly, at this stage of their development, angelfish are not expected to engage in territorial and/or reproductive behaviours, potential complicating factors that would influence their behaviour in the food choice tasks employed.

The holding aquaria were filled with dechlorinated tap water, continuously cleaned by external filters, and kept at 26 ± 1 °C using thermostat-controlled heaters. Each aquarium was illuminated by a 15-W white fluorescent light tube, and a L:D 12:12 h lighting regime was maintained with lights on at 0830 hours. Except from the front, the aquaria were lined on the outside with white cardboard, and provided with a gravel substratum. The fish were fed twice daily (morning and afternoon) on commercial food flakes.

### Experimental apparatus and stimuli

The only experimental aquarium (60 × 30 × 33 cm, length x width x depth) was maintained under the same conditions as the holding aquaria. Fish were recorded from above, and all exterior walls of the aquarium were lined on the outside with white cardboard to prevent the fish being influenced by external visual stimuli. As described before^6^, two transparent plastic partitions divided the aquarium into three compartments. Partitions were placed 25 cm from each lateral short side of the aquarium (see Fig. 1). In the center of each partition, a small rectangular guillotine window could be opened to allow fish to pass through between compartments. The central part of the smaller middle compartment constituted the start box (10 × 10 × 33 cm length × width × depth) from where the experimental fish were released at the start of the behavioral session (Fig. 1).

The two lateral compartments of the experimental aquarium were considered as the ‘home compartment’ and the ‘test compartment’ alternatively. In the latter, the visual stimuli were presented during testing. In the middle of the test compartment, a transparent plastic divider delimited two equally-sized halves: the ‘preference zones’ (10.5 × 15 cm, width × length; Fig. 1).

In each of the preference zones two quantities of discrete food items were simultaneously presented. The food sets remained visible during the test period and, to avoid any potential chemical cue, during tests food quantities were presented outside the experimental aquarium. Thus, only visual cues of the food items were accessible for the test fish. Food items were pasted on a 5 × 5 cm area at the terminal part of transparent plastic panels 4 cm from the bottom end and were positioned flush against the exterior end wall in the testing compartment at a distance of 10 cm apart from each other. Discrete food items were prepared by making a homogeneous mass with the flakes using some water. The mass was agglutinated, and circular pieces of different size, depending on the experiment, were obtained. A methacrylate mold sheet (0.1 cm thick) perforated with holes of different diameters were used, into which portions of the agglutinate were introduced to obtain food items with the size required. We emphasize that the food items were presented outside of the tank and thus experimental fish could not eat or smell them. We also note that angelfish often nibble at the food items and thus mouth size relative to food item size may not be a factor in eating/accessing the food items presented^45,72^.

To avoid potential discrimination based upon configuration of the food items in the sets, in some experiments for each contrast presented 12 different configuration patterns (spatial arrangement of the food items) were used, in such a way that for any pair of contrasts fish were presented with a different stimulus configuration (see Fig. 2).

### Procedure

The procedure was the same as that described previously^6^.

#### Habituation phase

Shoals of 10 angelfish were placed into the experimental aquarium 7 days before the experiments. During this phase fish could explore the three compartments and pass through the guillotine windows freely. Four food items were provided as discrete items (0.4 cm Ø) pasted onto four panels lowered into the water in each feeding session. The size of each item was big enough to allow feeding for more than one fish at a time. In fact, frequent observations (using a computer monitor) indicated that fish were distributed evenly, with 2–3 fish around each of the food items. The panels were uniformly distributed along the long walls of the aquarium and leaning against the walls (two panels on one wall and the other two on the opposite long wall of the aquarium). In this way, monopolization of food by potentially more dominant foragers, i.e., potential competition over food among conspecifics, was reduced. Aggressive behaviours or monopolization of items were rarely seen. Apparently, all fish were occupied with feeding and/or with trying to catch food. Angelfish can manoeuvre well when catching food. Typically, fish after pecking at an item, swam back a little before pecking on it again. In the meantime, another fish did the same, and so on. Likewise, the distribution of the food items throughout the aquarium and their location in the long walls (instead of in the short wall as during actual choice tests) prevented angelfish from associating the food with one specific location in the aquarium. Always 8–10 fish were in the experimental aquarium, since every second day new fish were transferred to replace those (two fish) that had been tested. The feeding schedule and amount of food available remained as in the holding aquaria.

#### Test phase

In the test phase, we examined the preference of the test fish in a spontaneous binary choice task. Before starting each trial, an opaque white partition identical to the transparent partitions, including the guillotine window, was superimposed over one of the transparent partitions. The one that was covered was counterbalanced between the two transparent partitions that separated the lateral compartments to prevent any lateral side bias. All 10 fish were kept in the compartment now separated by the opaque partition (home compartment) and the experimenter closed the opaque guillotine window, thus blocking the view of the other side of the aquarium. The transparent guillotine window, in the transparent partition separating the other compartment (the test compartment), was also closed by a transparent panel. While all fish were in the home compartment, the corresponding food items glued on each of two panels were placed in the external side of each of the preference zones of the test compartment. After a 3-min period, the experimenter opened the opaque white guillotine window of the home compartment and waited until one subject spontaneously swam through the window into the start box. Immediately that guillotine window was closed. Thus, we limited the entrance of only one subject into the start box, and the remaining subjects could not see what happened on the other side of the partition. After the subject had been in the start box for 30 s, during which it could see the two sets of food items through the transparent partition, we gently opened the transparent guillotine window to allow the fish to freely enter the test compartment to make the choice.

Tests took place in the morning at the usual feeding time (1000–1015 hours), thus subjects were not food-deprived, but they were sufficiently motivated to perform the task. A camera placed above the experimental aquarium recorded the behaviour of the subjects for 5 min. To control for possible side preferences, we also counterbalanced the left-right presentations of the stimuli across fish, and reverse the presentation of the sets between the lateral walls of the aquarium in a pseudo-randomized order. The order of presentation of each stimulus contrasts was also randomized across fish.

We recorded the first preference zone selected by the experimental angelfish, defined as the first preference zone in which the fish entered after leaving the start box. Also, we recorded the time spent (sec) in each preference zone. For each fish, we calculated an index to quantify preference for one stimulus or set over the other as the proportion of time spent in the preference zone close to the larger item or the set with the larger number of items relative to the total time spent in both preference zones. The frequency of entries to the preference zones, that is, the number of times fish entered to the preference zones, and the latency to enter the preference zones, defined as the time (sec) elapsed since the fish left the start box and entered one of the preference zones, were also recorded. Twelve naïve subjects were tested in each of the contrasts of each experiment.

Each fish was tested only once for a single pair of contrasts and one fish was tested per day. After having been tested, each subject was moved to a holding tank and fed. Likewise, the rest of the fish of the shoal were fed in the usual way in the experimental aquarium, after opening the guillotine windows. Every second day, after two fish had been tested and removed, two new fish were transferred to the experimental aquarium to maintain shoal size in the experimental aquarium relatively constant.

### Statistical analysis

In each experiment, tests for normality (Shapiro-Wilk test) and for equality of variance (Levene’s test) were performed on the data before analysis. If the data tested were not normally distributed, they were log transformed before the analyses to meet assumptions of parametric statistics (those of latency as well as group 4:1 size ratio of Experiment 1 in the frequency of entries, p ≤ 0.05). The proportions of time spent in the preference zones were normally distributed, and one sample t-tests were employed to investigate whether the observed preference index was significantly (p ≤ 0.05) different from chance (index = 0.5). The Holm-Bonferroni sequential correction method was employed to correct for type I error resulting from multiple comparisons. Effect size for significant results was calculated using Cohen’s *d*. One-way ANOVA for independent samples was used to analyze the effect of the contrasts on preference. In case of a significant effect, it was followed by a Tukey Honestly Significant Difference (HSD) post hoc multiple comparison test. Effect size for significant results was calculated using partial eta-squared (${\eta }_{p}^{2}$). Linear regression analysis was performed to quantify the strength of the relationship between the proportion of time (dependent variable: preference index) test fish spent in proximity of the larger-sized food item and the ratio between the contour length of the items (independent variable or explanatory/predictor variable). Binomial tests comparing the number of fish initially choosing one stimulus or the other were used for each combination of stimuli, and frequency and latency scores were analyzed using paired t tests. All tests are two tailed. Statistical analyses were performed using SPSS Statistics 23.0.

Subjects that did not enter both preference zones at least once were excluded from the analysis and were replaced by others. In Experiment 1, five fish (7%) in Experiment 2, two fish (5.5%) and in Experiment 3, one fish (4%) were excluded and replaced.

### Experiment 1

Although we used a paradigm whose methodological details have been recently established^6^, first we tested some basic performance aspects of angelfish in the paradigm. Thus, in Experiment 1, we presented subjects with a choice between two single food items of identical size (1 vs. 1; 0.4 cm Ø), placed in the test compartment and flush against the exterior wall aquarium (i.e., fish could not eat the items) (Fig. 1). The aim was to determine whether angelfish responded to the task and showed any evidence of side bias. This experiment also served as control and was meant to establish a baseline for the response. Subsequently, we examined whether increasing the contour length (diameter) of one of the circular single food items relative to the other induces differential responses (Fig. 2), including a preference for the larger food item. Different size ratios (larger:smaller item size) were employed by presenting circular food items with diameter of 1.6 cm vs. 0.4 cm (4:1 ratio), 1.2 cm vs. 0.4 cm (3:1 ratio), 0.8 cm vs. 0.4 cm (2:1 ratio), 0.6 cm vs. 0.4 cm (1.5:1 ratio), and 0.53 cm vs. 0.4 cm (1.33:1 ratio; see Fig. 2).

### Experiment 2

In Experiment 2, we manipulated the contour length (diameter) of the circular food items in the numerically different sets in such a way that each item in the numerically smaller set had larger sized items compared to items in the numerically larger set, leading to equal total visible contour length for the two sets in each contrast (Fig. 2). Since subjects in the start box (prior to the start of the tests) could see the two contrasted food sets, it is assumed that they were informed about the profitability of each set. The aim was to investigate whether preference for number of food items or for size of the food items takes priority in food decision-making in angelfish. Thus, we presented numerically different sets of food items (4 vs. 2, 3 vs. 2 and 4 vs. 3) that contained different ratios of the item sizes, i.e., different diameter ratios of the items of 2:1, 1.5:1 and 1.33:1, respectively (Fig. 2), but we equated the overall perimeter (contour length) of the items in the contrasted two sets. More specifically, in the numerical contrast 4 vs. 2, each item of the 4-item set had 0.3 cm Ø and each item of the 2-item set had 0.6 cm Ø; in the contrast 3 vs. 2, each item of the 3-item set had 0.4 cm Ø and each of the 2-item set had 0.6 cm Ø; and in the numerical contrast 4 vs. 3, each item of the 4-item set had 0.4 cm Ø and each item of the 3-item set had 0.53 cm Ø.

### Experiment 3

In Experiment 3, we tested whether the preference for the larger item size can lead to suboptimal responding. We tested this possibility by conflicting visible contour length of the food items presented (i.e., number of food items) with food item size by presenting one larger food item in the numerically smaller set whose items had smaller overall size (contour length) (Fig. 2). In a similar task, a suboptimal response has been found at least in fish^4^ and primates^40,43^, whereby individuals were found to prefer the set containing a single larger sized food item despite that such a set offered less total food quantity. Thus, items in the numerically larger set were all of 0.3 cm Ø, but whereas one of the items in the numerically smaller set was larger, 0.6 cm Ø, the other was smaller: 0.3 cm Ø. The numerical contrasts presented were 4 vs. 2 and 6 vs. 2, with a total diameter of the larger sets being 1.2 cm and 1.8 cm, respectively, whereas that of the smaller set that contained the larger-sized item was in both contrasts 0.9 cm. Therefore, the overall ratio of contour length of these sets was 1.33:1 and 2:1, respectively (Fig. 2).

### Ethics statement

The experiments complied with the current law of the country (Spain) in which they were performed and were approved by the Ethics Committee of the University of Oviedo (permit ref.: 13-INV-2010). The experiments involved behavioural observation with no invasive manipulation on the fish, which remained healthy over the course of the experiments. All fish were returned to the supplier at the end of the study.

## Data Availability

The data set generated during the present study are available from the corresponding author on reasonable request.
